# SnoRNAs: Exploring Their Implication in Human Diseases

**DOI:** 10.3390/ijms25137202

**Published:** 2024-06-29

**Authors:** Waseem Chauhan, Sudharshan SJ, Sweta Kafle, Rahima Zennadi

**Affiliations:** Department of Physiology, University of Tennessee Health Science Center, 71 S. Manassas St., Memphis, TN 38103, USA; wchauhan@uthsc.edu (W.C.); ssetraja@uthsc.edu (S.S.); kaflesa@mail.uc.edu (S.K.)

**Keywords:** small non-coding nucleolar RNA, small nucleolar ribonucleoproteins, snoRNA biogenesis, RNA processing, human pathologies

## Abstract

Small nucleolar RNAs (snoRNAs) are earning increasing attention from research communities due to their critical role in the post-transcriptional modification of various RNAs. These snoRNAs, along with their associated proteins, are crucial in regulating the expression of a vast array of genes in different human diseases. Primarily, snoRNAs facilitate modifications such as 2′-O-methylation, N-4-acetylation, and pseudouridylation, which impact not only ribosomal RNA (rRNA) and their synthesis but also different RNAs. Functionally, snoRNAs bind with core proteins to form small nucleolar ribonucleoproteins (snoRNPs). These snoRNAs then direct the protein complex to specific sites on target RNA molecules where modifications are necessary for either standard cellular operations or the regulation of pathological mechanisms. At these targeted sites, the proteins coupled with snoRNPs perform the modification processes that are vital for controlling cellular functions. The unique characteristics of snoRNAs and their involvement in various non-metabolic and metabolic diseases highlight their potential as therapeutic targets. Moreover, the precise targeting capability of snoRNAs might be harnessed as a molecular tool to therapeutically address various disease conditions. This review delves into the role of snoRNAs in health and disease and explores the broad potential of these snoRNAs as therapeutic agents in human pathologies.

## 1. Introduction

Research over the past decade has revealed that non-coding regions of the human genome significantly influence gene expression, affecting disease development beyond the role of protein-coding genes. It has now become apparent that RNA in the nucleus undergoes extensive post-transcriptional modifications specific to nucleosides. These modifications on RNA molecules are essential for key cellular processes like RNA maturation, stability, ribosome assembly, translation, and transfer RNA recognition [1,2,3,4,5,6,7]. Nucleoside modifications exhibit evolutionary conservation from bacteria to humans [1,2,3,4,5,6,7], and they involve RNAs transcribed from non-coding regions of the genome. This prompted researchers to gain an understanding of how non-coding RNA (ncRNA) biogenesis is regulated and its role in post-transcriptional modifications. Small nucleolar RNAs (snoRNAs), a type of ncRNAs, typically 60–300 nucleotides long, mainly accumulate in nucleoli or nucleoplasmic Cajal bodies, which are rich in RNA and RNA binding proteins (RBPs). They guide the RNA-dependent modifications and maturation of ribosomal RNAs (rRNAs), small nuclear RNAs (snRNAs), and other cellular RNAs [8] by forming small nucleolar ribonucleoprotein (snoRNP) complexes with RBPs and other proteins. However, some snoRNAs function as RNA chaperones in pre-rRNA maturation, not as guides for RNA modification [9]. 

snoRNAs were first discovered in the 1960s during studies on ribosome biogenesis. Initially, they were identified as small RNA molecules associated with the nucleolus, a subnuclear structure responsible for ribosomal RNA (rRNA) synthesis and assembly [10,11]. The understanding of snoRNAs expanded significantly in the 1980s and 1990s when it was discovered that they guide the chemical modifications of rRNAs, such as 2′-O-methylation and pseudouridylation, which are essential for the proper assembly and function of ribosomes [12,13,14]. Further research revealed that snoRNAs are involved in modifying other types of RNA, including small nuclear RNAs (snRNAs), highlighting their broader role in RNA processing and the regulation of gene expression.

Primarily, snoRNAs are divided into two classes: C/D box snoRNAs and H/ACA box snoRNAs [15]. C/D box snoRNAs guide the methylation of ribose at the 2′-hydroxyl group, while H/ACA box snoRNAs facilitate the conversion of uridines into pseudouridines (Ψ) through the pseudouridylation of nucleotides [9]. In general, mammalian cells contain hundreds to thousands of copies of various expressed snoRNAs [16,17]. SnoRNAs were among the first ncRNAs sequenced using RNase-mapping in 1979 [18], soon after the advent of Sanger sequencing [19].

## 2. Structure of snoRNAs

**Figure 1:**
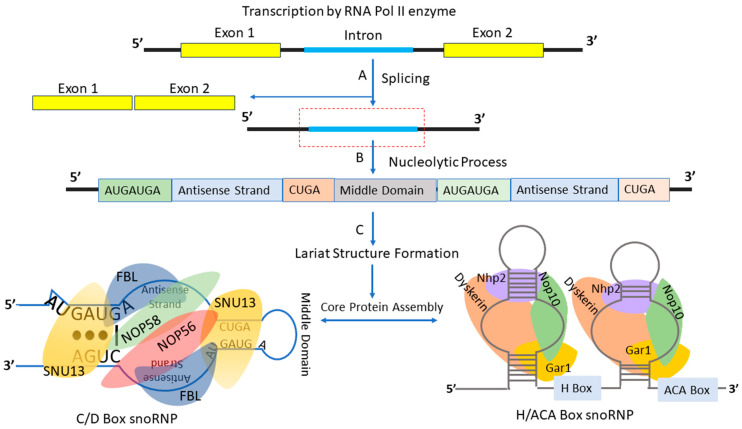
**Diagrammatic representation of snoRNA biogenesis.** (A) After the transcription process, newly formed mRNA undergoes a splicing process to remove the introns. (B,C) These introns undergo further nucleolytic processing, then lariat structure formation evolves as either C/D box snoRNAs or H/ACA snoRNAs. (C) The snoRNAs specifically bind to the SNU13 (aka 15.5K), NOP56, NOP58 and FBL proteins to become C/D box snoRNPs, while the assembly of H/ACA snoRNPs involves core proteins such as Nap57, Cbf5p (dyskerin), and GAR1.

The mammalian genome contains around 400 distinct snoRNA sequences, essential for 2′-O-methylation, pseudouridylation, and processing various cellular RNAs [20]. Both C/D and H/ACA boxes exhibit a high degree of structural conservation across species, ranging from humans to archaea [21]. Eukaryotic C/D box snoRNAs are usually 70 to 120 nucleotides long [22]. Box C (RUGAUGA) and box D (uCUGA) exist as pairs, forming box C/C’ and box D/D’. The C’ and D’ boxes show some degeneracy. Box C/D snoRNAs have a hairpin shape with a large internal loop and C/C’, D/D’ motifs. They form a “kink-turn” structure and have a 7–21 nucleotide antisense element (ASE; also called guide region) complementary to target RNAs, located upstream of the D/D’ boxes. Box C/D snoRNAs associate with four distinct partner proteins: fibrillarin (FBL), Nop56, Nop58, and 15.5K (snu13p)/NHPX, forming stable, active snoRNP complexes (Figure 1 below) [23,24]. The assembly of snoRNA with its proteins starts with the k-turn and snu13p interaction, leading to a structure that attracts FBL and Nop58, followed by other proteins [21,23]. FBL is structurally similar to S-adenosyl methionine (SAM)-dependent methyltransferase [25]. The associated proteins shield snoRNAs from exonuclease digestion and influence their nuclear placement [20]. The typical H/ACA snoRNA structure, however, includes two hairpin structures for identifying rRNA pseudouridine sites, featuring an H box and a 3′ end ACA box [26]. H/ACA snoRNAs, typically 60–75 nucleotides long, have pseudo-uridylation pockets for isomerizing uridine residues on substrate RNA [27]. Unlike box C/D snoRNAs, H/ACA snoRNPs assembly involves core proteins like Nap57, Cbf5p (dyskerin), and GAR1 (Figure 1 below) [28,29]. Alternatively, small Cajal body specific RNAs (scaRNAs) feature a lengthy UG repeat in box C/D or an extra CAB box (UGAG motif) in box H/ACA [4,30]. scaRNAs are a class of snoRNAs that are specifically localized to Cajal bodies within the cell nucleus. These RNAs guide the chemical modifications of small nuclear RNAs (snRNAs), particularly the 2′-O-methylation and pseudouridylation processes, which are essential for the biogenesis and function of spliceosomal snRNAs; they are also needed for modification on ribosomal RNAs (rRNAs) in the Cajal body [31,32]. ScaRNAs play a crucial role in the maturation of snRNAs and, consequently, in the regulation of pre-mRNA splicing [32]. Orphan snoRNAs, lacking complementarity to rRNAs or snRNAs, also exist, but with largely undetermined functions.

The Human Gene Nomenclature Committee named C/D box snoRNAs SNORDs, each with a unique number. Previously, small nuclear RNAs, rich in Uracil, were labeled with U and a number, like U1, U2, and U3. Some, like U3, were later identified as SNORDs. Newly discovered snoRNAs received sequential numbers without distinguishing between C/D and H/ACA types. Occasionally, these numbers are significant, like U27 (now SNORD27), which is predicted to direct the methylation of A27 in 18S rRNA [33].

## 3. Biogenesis of snoRNAs

SnoRNAs arise as byproducts of RNA processing, often originating from intronic regions of 5′-uncapped mRNA [34]. SnoRNA generation is linked with splicing, debranching, and trimming processes (Figure 1) [9,35,36]. However, a subset of these small non-coding RNAs originates from intergenic regions with independent promoters marked by a 2,2,7-trimethylguanosine (TMG) cap structure [37]. Key snoRNAs like U3, U8, and U13, involved in pre-rRNA endonucleolytic processing, are the product of RNA polymerase II. Genes, hosting snoRNAs in introns, are termed host genes [15,38], numbering over 200. Some host genes are non-protein-coding with short, variable open reading frames [35], initially deemed non-functional until recent discoveries challenged this notion [39,40].

RNA polymerase II (Pol II) transcribes snoRNA genes similarly to mRNAs [41]. However, unlike mRNAs, which are exported from the nucleus, snoRNAs remain inside it [37,42]. This difference may be due to the lack of mRNA-like structural features in snoRNAs, such as the m^7^G cap bound by the cap-binding complex (CBC) and the poly(A) tail, which aid in mRNA export. These elements are removed during snoRNA maturation. For instance, in *S. cerevisiae*, snoRNAs with a 5′ end like mRNAs are transported out of the nucleus [43]. In humans, snoRNAs are mainly found within introns, segments removed from protein-coding genes during mRNA splicing. Upon liberation during splicing, introns usually form a lariat structure, which is then debranched or linearized by a debranching enzyme (Figure 1). SnoRNA processing is consistent across classes and organisms: pre-snoRNAs are trimmed by ribonucleases at the 3′ end or both the 5′ and 3′ ends. Unprotected regions at the 5′ and 3′ ends are removed. In situations where the Transcription Start Site (TSS) defines the mature 5′ end, the snoRNA’s cap remains and undergoes post-transcriptional modifications (Figure 1).

snRNAs have been classified into two primary groups based on their intranuclear localization [44,45]. The snRNAs U1, U2, U4, U5, and U6 are primarily located in the nucleoplasm [45], except U3, which is in the nucleoli and classified as a snoRNA [46,47,48]. U3 is notable for its evolutionarily conserved motifs, C (RUGAUGA) and D (uCUGA), and its association with the nucleolar protein FBL [49,50]. In contrast, snoRNAs U17, E2, and E3 represent a different class with a distinct “hairpin-hinge-hairpin-tail” structure [51,52,53,54] and conserved box H (AnAnnA) and ACA motifs [51].

a. *Processing the snoRNA 3′ end in humans*

The processing of an RNA precursor’s 3′ end, alongside transcription termination, is critical in determining its fate as mRNA or ncRNA. In eukaryotic cells, mRNA stability is enhanced by mRNA-specific transcription termination and 3′ end processing, executed by cleavage and polyadenylation (CPA) machinery. This machinery includes RNA endonuclease, the cleavage and polyadenylated specificity factor (CPSF)-73, and poly(A) polymerase PAP, supported by cleavage stimulation factor (CstF) and cleavage factors I and II (CFIm and CFIIm with PCF11) [55]. On the other hand, shorter non-coding genes employ different mechanisms that connect transcription termination with 3′-5′ degradation [56,57].

In human cells, only a few snoRNA genes, such as U3, U8, U13, and telomerase snoRNA, are expressed independently [37,58], and their biogenesis is not fully understood. They may share mechanisms with other short non-coding RNAs (ncRNAs). Unlike in yeast, human cells lack the Nrd1-Nab3-Sen1 (NNS) complex, so the termination of short ncRNAs, including snoRNA-like snRNAs, enhancer RNAs (eRNAs), and promoter upstream transcripts (PROMPTs), primarily depends on CPA factors and possibly the 3′ end processing integrator complex. This complex is vital for cleaving snRNAs and eRNAs, thus facilitating their release from RNA polymerase II during transcription termination [59,60,61].

The termination of certain ncRNAs requires the recruitment of a protein named ARS2. ARS2 operates similarly to the yeast NNS complex, linking transcription termination with 3′ end processing [62,63,64]. ARS2, by binding to the cap-binding complex (CBC), forms the CBC-ARS2 (CBCA) complex. This leads to its attachment to RNA, thereby initiating the termination of snRNAs, eRNAs, and PROMPTs near the transcription start site [63,64]. The CBC is believed to bind various m^7^G-capped RNAs, including mRNA precursors and mature forms, long non-coding RNAs, non-adenylated histone RNAs, and spliceosomal snRNA precursors. It is also linked to m^7^G-capped snoRNAs. However, the involvement of CBCA in snoRNA 3′ end processing is uncertain.

Research shows that removing ARS2 and CBC does not impact the transcription termination of U8 snoRNA [63]. The 3′ end processing of independent snoRNAs might involve the exosome and CBCA. CBCA could interact with the zinc-finger protein ZC3H18, linking to the NEXT (Nuclear Exosome Targeting) complex to create the CBC-NEXT assembly, which then connects to the RNA exosome [63,65]. For other short ncRNAs, PHAX (involved in pre-snRNA/snoRNA transport) and ZC3H18 compete to bind with CBC. This competition is significant, as it prevents the NEXT complex from binding to RNA precursors [66]. NEXT can load onto 3′ unprocessed snoRNAs independently of CBC, aided by the RNA-binding protein RBM7 [65,66].

Human snoRNAs are processed from spliced introns or precursors, either exonucleolytically or through endonucleolytic cleavages [67,68,69,70]. RBM7/NEXT and the exosome can lead to a complete degradation of certain pre-snoRNAs. Poly(A)-specific ribonuclease (PARN) and a target of EGR1 protein 1 (TOE1) are known to aid in maturing ncRNAs like snoRNAs, scaRNAs, and telomerase RNA component (TERC), which have motifs similar to H/ACA box scaRNAs [71,72,73]. When both PARN and TOE1 are impaired, scaRNAs significantly decrease, causing snRNA pseudouridylation issues [73]. PARN and TOE1, by removing oligo(A) tails, protect ncRNAs from decay, like protein de-ubiquitination [74].

b. *Processing the snoRNA 5′ end in human*

In human cells, pre-snoRNA undergoes 5′ end processing where the m^7^G cap is transformed into a 2,2,7-trimethylguanosine (TMG) cap, potentially by the action of a nucleolar trimethylguanosine synthetase, Tgs1 [75]. However, the details of this process in snoRNA biogenesis are not well understood. Knocking down Tgs1 in human cells disrupts Cajal bodies, but it is unclear if this directly affects snoRNA or snRNA synthesis, despite some of their formation stages occurring within these structures [76].

Knowledge about the enzymes involved in the 5′ end trimming of intronic snoRNAs in vertebrates is limited. It is known that unspecified 5′-3′ exonucleases perform the excision and embranchment of introns, which are essential for the processing reaction [67,68]. The reasons why snoRNAs typically lack m^7^G caps, possibly related to their genomic organization or 5′ end processing, are under investigation. The presence of m^7^G caps, common in mRNAs, might influence snoRNA function and stability, thereby impacting ribosome biogenesis and translation regulation. Different maturation pathways for polymerase II-generated snoRNA and mRNA transcripts are determined by signals in the nascent transcripts and the sequence of processing events.

c. *snoRNA-derived RNAs (sdRNAs)*

snoRNAs are processed into smaller fragments, some of which are not merely degradation products [77]. This is supported by the conservation of sdRNA expression across species, the derivation of highly abundant sdRNAs from weakly expressed snoRNAs, and the specific stabilization of snoRNA-derived fragments. sdRNAs can be categorized by their origin and length: H/ACA box snoRNAs generate 20–24 nt fragments mainly from the 3′ end, while C/D box snoRNAs produce fragments greater than 26 nt and 17–19 nt mainly from the 5′ end. Over half of all snoRNAs produce these smaller fragments, suggesting potential novel roles, although the functions of many sdRNAs remain unknown [77]. sdRNA fragment expression often changes in pathological conditions like cancer [78,79], but whether they drive the pathology or merely indicate disease progression is still unclear.

d. *RNA A-to-I editing*

The RNA pool of many metazoan organisms is subject to A-to-I (adenosine-to-inosine) RNA editing. The specific catalysis of double-stranded RNA (dsRNA) adenosine deaminase (ADARs) to convert adenosine to inosine is common for RNA editing [80]. However, this editing is not random and only selective adenosines are replaced [81]. Furthermore, a recent study proposed that snoRNAs also undergo RNA A-to-I editing after binding to ADAR1. Two C/D box snoRNA sequences, SNORD49A and SNORD49B, have been found in a novel snoRNA-related lncRNA called lnc-SNO49AB, and these lncRNAs are highly expressed in samples from leukemia patients, suggesting their role in leukemia progression. It was also shown that lnc-SNO49AB may bind directly to the adenosine deaminase that was operating on RNA 1 (ADAR1), causing it to homodimerize and then exhibit a high level of RNA A-to-I editing activity [80,82].

## 4. Regulation of snoRNAs Expression and Activities

The regulation of snoRNA expression and their activities may be achieved either through the direct post-transcriptional modification of snoRNAs or via regulating the expression or activities of their protein partners (FBL, Nop58, Nop56, and 15.5K). However, very few reports have stated regulation of snoRNAs post-transcriptionally. Fused with Liposarcoma (FUS), one of the three members of FET family of proteins [FET is named after FUS, EWS (Ewing Sarcoma, and TAF15 (TATAbinding associated factor15)], has DNA and RNA binding affinity and is predominantly involved in transcription, splicing and alternative splicing [83,84]. Recently, a research group has used FUS knockout (KO) HEK293T and SH-SY5Y cell lines and reported that SNORD48, SNORD90, and SNORD100 are upregulated in SH-SY5Y FUS KO cells, presuming that FUS is affecting snoRNAs maturation, although the mechanism is still obscure [85]. Additionally, they have also found a changed expression of FBL, Dyskerin and Nop56 proteins in FUS KO cells [85]. In addition, Peroxiredoxin 1 (Prx1) can also bind with RNA. However, this protein is a reactive oxygen species (ROS) scavenger, but this antioxidant functions according to the reduced or oxidized state of its conserved catalytic cysteine residues. When it is in the reduced state, Prx1 regulates the expression of a set of snoRNAs post-transcriptionally [86].

## 5. RNA Modifications by snoRNAs

RNA molecules experience several post-transcriptional modifications during maturation. Traditionally, snoRNAs are known for modifying rRNAs and snRNAs through pseudouridylation and methylation. However, recent studies suggest that snoRNAs might also modify different RNA types through N4-acetylcytidine (ac4C) alterations [8]. These changes regulate alternative splicing similar to miRNAs function; such changes are crucial for maintaining cellular health. In addition, stable long non-coding RNAs (lncRNAs) can be generated by collaborating with snoRNA processing to produce snoRNA-ended lncRNAs (sno-lncRNAs) and 5′ snoRNA-ended, 3′-polyadenylated lncRNAs (SPAs) [87].

- *Pseudouridylation of RNAs*

**Figure 2:**
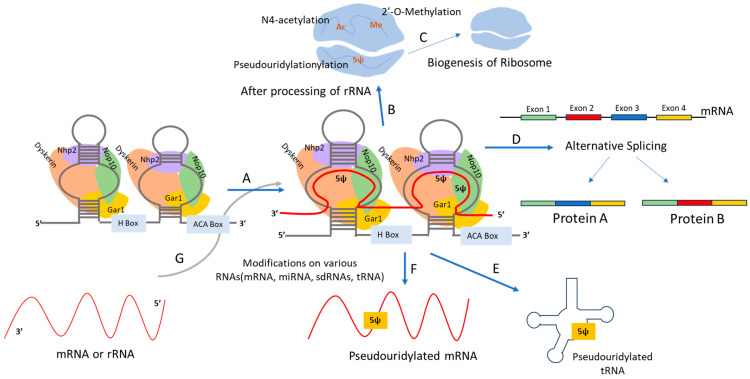
**Depicting H/ACA snoRNP modifying various RNAs.** (A,G) The H/ACA box snoRNP formed after the snoRNA is associated with multiple proteins (e.g., Nhp2, Nop10, Dyskerin and Gar1), are actively involved in the modification of various RNAs (miRNA, tRNA, mRNA, rRNA or/and sdRNA). (B,C) These H/ACA box snoRNAs are also involved in modifications (N4-acetylation, 2′-O-methylation and Pseudouridylation) on rRNA, and then help in the biogenesis of ribosomes. (D) Alternative splicing of mRNA is one of the important functions of these snoRNAs as well. (E,F) These H/ACA box snoRNAs are in addition actively involved in the pseudouridylation of mRNAs and tRNAs and help in their maturation.

Pseudouridine (Ψ) is the most abundant [88] and functionally important modification in cellular RNAs, including tRNAs, rRNAs, and snRNAs [89]. Notably, about 8% of uridine residues in human rRNAs are converted to Ψ through a complex mechanism. This conversion in eukaryotes, especially in rRNAs and snRNAs, is primarily performed by box H/ACA class ribonucleoproteins—the most intricate known pseudouridylases [90,91]. These proteins, including Nop10, Gar1, NhP2, and dyskerin, catalyze the isomerization of uridine to Ψ and are highly conserved [92] (Figure 2).

In 1997, researchers discovered that box H/ACA snoRNAs act as guide RNAs, targeting uridine in rRNAs for pseudouridylation [93]. Each box H/ACA snoRNA has a distinct hairpin-hinge-hairpin-tail structure, with conserved H and ACA boxes in the hinge and tail, respectively. These structures create loops complementary to targeting RNA sequences, positioning uridine at the hairpin’s upper stem for pseudouridylation via Watson–Crick base pairing (Figure 2) [89,93,94]. Box H/ACA snoRNAs are also implicated in the pseudouridylation of snRNAs and mRNAs [31,95], with snRNA pseudouridylation occurring in Cajal bodies instead of nucleoli [31]. A study found that artificial box H/ACA snoRNAs, when targeted to pre-mRNA in *Xenopus oocytes*, caused pseudouridylation at specific sites, leading to splicing defects [96]. This is crucial because the pseudouridylation of stop codons (UAA, UAG, UGA), traditionally recognized by the release factor to signal the termination of the translation process [97], alters their properties, making them unrecognizable to the release factor, resulting in continuous translation and potentially different protein behavior [89,95].

b. *2′-O-methylation*

**Figure 3:**
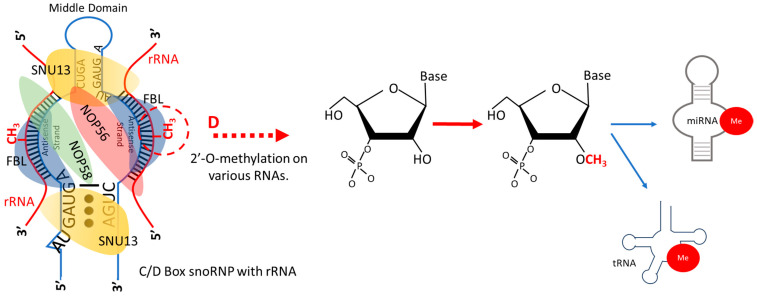
**Box C/D snoRNA-guided 2′-O-methylation of various RNAs.** Once the box C/D snoRNAs are bound to the SNU13 (aka 15.5K), NOP56, NOP58 and FBL proteins to become C/D box snoRNPs, these proteins are guided by snoRNAs to target RNAs and bind to a complementary sequence on the RNA target through a short antisense element. The RNA target then undergoes 2′-O-methylation at a specific site mediated by FBL. Nm modification occurs on various RNA types.

Besides pseudouridylation, another key RNA modification is 2′-O methylation, also known as Nm modification. In Nm modification, any nucleotide (denoted as “N”) undergoes methylation, where a methyl group (-CH3) attaches to the 2′ hydroxyl group of the fifth ribose sugar in the RNA’s backbone. This methylation can happen on any nucleotide base, as they all have ribose sugar. C/D box snoRNAs target specific RNA sequences, binding through their antisense elements upstream of the D/D′ boxes. They form a duplex at the modification site, where RNA-binding proteins then modify a specific nucleoside [15]. The enzyme FBL is the one responsible for 2′-O-ribose methylation on RNA, guided by C/D box snoRNA (Figure 3). This modification is widespread and conserved, appearing in various RNA types such as tRNA, rRNA, snRNA, sncRNAs, miRNAs, mRNA, siRNAs, and piRNAs [98,99,100,101,102,103,104]. It significantly changes the RNA’s biophysical properties, increasing hydrophobicity and thermodynamic stability and stabilizing its helical structure, which protects against nucleases. Additionally, it alters the RNA’s tertiary structure and hydrogen bonding, impacting RNA–protein interactions [98,99,100,101,102,103,104].

c. *N4-acetylcytidine (ac4C) modification*

**Figure 4:**
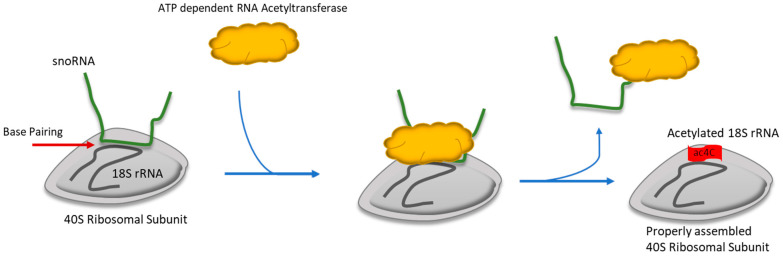
**Acetylation of 18S ribosomal RNA.** Acetylation is one of the other important modifications required for the proper assembly of the ribosome or ribosomal biogenesis. In this figure, it is evident that snoRNA makes the Watson–Crick base pairing with 18S rRNA but prior to the interaction with RNA acetyltransferase. RNA acetyltansferase helps then the 18S rRNA to be navigated, prior to acetylate cytidine residues.

ac4C modification, essential for maturing rRNAs, tRNAs, and mRNAs, is highly conserved [105,106,107]. ATP-dependent acetyltransferase enzymes, like NAT10 in mammals and Kre33 in yeast, catalyze this modification [105,106,107]. Notably, NAT10 specifically adds the acetyl group for ac4C at position 1842 in mammalian 18S rRNA [107]. This enzyme, homologous to Rra1p (ribosomal RNA cytidine acetyltransferase 1), is an ATP-dependent RNA acetyltransferase [107]. In eukaryotic 18S rRNA, modified cytidines in helix 34 aid in translation, and those in helix 45 contribute to the ribosomal decoding site [105]. NAT10, assisted by snoRNA, performs ac4C modification on these cytidines [105,108,109]. During acetylation, NAT10 and Kre33 work with box C/D snoRNAs snR4 and snR45 [108], which helps locate the specific acetylation sites in 18S rRNA through base pairing [108] (Figure 4).

## 6. Regulation of mRNA and Long Non-Coding RNA (lncRNA) Splicing and Editing by snoRNAs

Recent studies have revealed new functions for snoRNAs beyond their traditional role in ribosomal biogenesis and RNA modifications. These include their involvement in the alternative splicing of mRNAs [33] and influencing chromatin structure. Notably, SNORD115, an orphan snoRNA [8], is involved in the alternative splicing of the serotonin receptor 2c (Htr2c) mRNA, creating a variant receptor isoform [110]. Additionally, it competes with Adenosine deaminases acting on RNA (ADAR) enzymes [111], affecting amino acid changes in exon 5 that influence receptor signaling [112]. Furthermore, SNORD115 targets five additional splicing sites [113], and SNORD116, found at the same chromosomal location, also contributes to splicing regulation [114]. It has also been evidenced that SNORD27 influences the splicing of the E2F7 transcription factor through competing with U1 snRNP [33], while SNORD88C affects the splicing of fibroblast growth factor receptor 3 by blocking cryptic splice sites [115].

Similar to typical mRNAs, most lncRNAs are also spliced and possess m7G caps and 3′ poly(A) tails. Some intronic lncRNAs have snoRNAs at both ends. The sno-lncRNAs have been identified in humans, rhesus monkeys, and mice, and show tissue- and species-specific expression patterns [116,117,118]. Typically, excised introns are debranched and degraded after splicing. However, when an intron contains two snoRNA genes, the formation of box C/D snoRNPs at the ends protects the intronic sequences from degradation, resulting in sno-lncRNAs [116]. Mutagenesis analyses have shown that the C box at the 5′ end and the D box at the 3′ end are crucial for sno-lncRNA processing [116].

In Prader–Willi syndrome (PWS), a neurodevelopmental disorder, the genomic region encoding several of the mostabundant box C/D snoRNA-ended sno-lncRNAs (ranging from 1000 to 3000 nts) is deleted [116]. These sno-lncRNAs can be precipitated by Fibrillarin, a key component of box C/D snoRNPs. Furthermore, a purified sno-lncRNA containing binding sites for the MS2 coat protein can co-purify with both Fibrillarin and the 15.5K protein [116,119].

## 7. Chromatin Remodeling by snoRNAs

Moreover, recent research links snoRNA-related proteins to chromatin remodeling and transcription [120,121], suggesting new roles for snoRNAs. These proteins interact with essential components of these processes or enhance gene expression. In yeast, Rvb1 and Rvb2, which are like human snoRNP proteins p55 and p50, are key parts of a chromatin remodeling complex [122]. Rvb1p and Rvb2p are highly conserved helicase subgroup proteins in eukaryotes, belonging to the AAA^+^ family of ATPases. Studies show that Rvb2 is involved in the early stages of snoRNP biogenesis, possibly linking snoRNA synthesis with snoRNP assembly and localization [123]. Notably, p50 and p55 proteins form associations in both yeast and human cells [124]. Additionally, proteins from rat and human cells have shown opposing polarity DNA helicase activities in in vitro experiments [124,125].

In plants, MARBP-1 and MARBP-2, are believed to have diverse roles in both chromatin organization and ribosome biogenesis [126], are similar to yeast nucleolar proteins Nop56p and Nop58p, which are crucial for ribosome biogenesis. This suggests that snoRNA-binding proteins might be involved in organizing chromatin and related processes, possibly in collaboration with snoRNAs. However, direct evidence of snoRNA involvement in these processes is still lacking, and these proteins may function independently of RNA. Future research is needed to clarify the role of snoRNAs in chromatin-related activities.

## 8. Implications of snoRNAs in Human Pathologies

a. - *Role of snoRNAs in regulating normal and malignant hematopoiesis*

Hematopoiesis is the process of blood cell formation, occurring primarily in the bone marrow. All blood cells originate from hematopoietic stem cells (HSCs) [127]. They can self-renew and evolve into multipotent progenitors, then differentiate and mature into functional blood cells. Maintaining a balance between self-renewal and differentiation is crucial for hematopoietic equilibrium in an organism’s life. Disruptions in this balance can lead to hematological disorders due to abnormal HSC behaviors [128]. Ribosomes are key regulators in maintaining the balance between HSC self-renewal and differentiation [129]. The progression of HSCs involves regulating the translation of specific mRNA molecules, mainly through rRNA and tRNA. These rRNA and tRNA undergo modifications controlled by snoRNAs [15]. A marked increased expression of snoRNAs contained in the *DLK-DIO3* locus was noted in acute promyelocytic leukemia, although their contribution to leukemogenesis is unknown [130]. This locus contained about 41 maternally expressed snoRNAs. Their expression was highest in CD34 cells but rapidly decreased with granulocytic differentiation, becoming nearly absent in mature neutrophils [131]. The expression of these snoRNAs was also markedly reduced in B cells and T cells [131]. Over 80 paternal snoRNAs in the SNURF/SNRPN locus showed a similar, but distinct, expression pattern. They were high in CD34 cells, decreasing after granulocytic differentiation, but remained, in contrast, high in B and T cells [131]. Such a locus contains two large orphan CD box snoRNAs, SNORD115 and SNORD116, showing high expression in CD34 cells but reduced expression during myeloid differentiation [131]. Loss of SNORD116 is notably important to the pathogenesis of Prader–Willi syndrome [132,133]. The genetic alterations that specifically target snoRNAs in AML were suggested to be uncommon [131].

**Figure 5:**
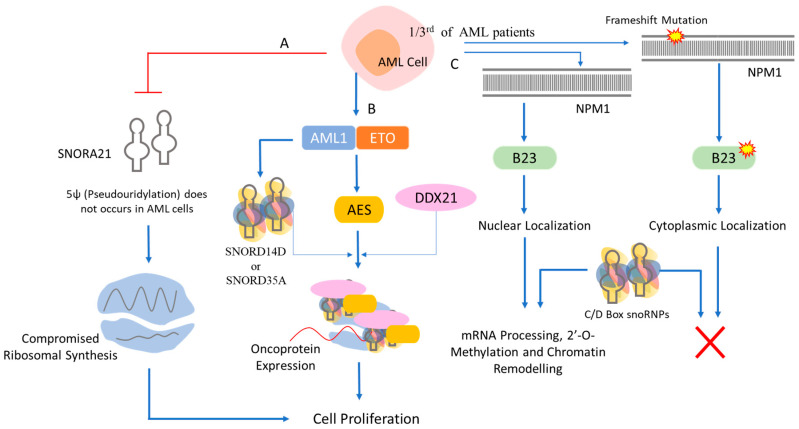
**snoRNAs with associated proteins participate in multiple cellular functions including cell proliferation in AML.** (A) AML cells lack SNORA21 and thereby do not show pseudouridylation that hampered the ribosomal synthesis needed for regulatory proteins. (B) AML cells also show chromosomal translocation t(8;21) causing chimeric protein (AML1-ETO) formation. AES shows oncogenic activity of this chimeric protein after the association with snoRNPs via interaction with DDX21. (C) Approximately, 30% of AML patients shows frameshift mutation in nucleophosmin (NPM1) protein, B23, which is destined to be in the nucleus but after frameshift mutation, B23 protein localizes in the cytosol. Thus, without B23 protein in the nucleus, C/D box RNPs are unable to process modification of the mRNA and chromatin remodeling.

In AML, the chimeric protein, AML1-ETO, is generated by chromosomal translocations, t(8;21) [134,135]. This protein acts as an oncogene, primarily promoting the self-renewal of hematopoietic progenitor cells [136]. Its activity relies on the expression of AES (groucho-related amino-terminal enhancer of split), which functions by inducing snoRNA/RNP formation via interaction with the RNA helicase DDX2 [136] (Figure 5). The deletion of C/D box snoRNAs SNORD14D or SNORD35A with a concomitant loss of rRNA 2′-O-methylation—both key factors in leukemic stem cell activity—leads to decreased potential for leukemia self-renewal. suggesting that the induction of snoRNA/RNP function constitutes an important pathway in leukaemogenesis [136]. Although the complete mechanism remains elusive, snoRNAs are associated with 2′-O-methylation and influence the translation efficiency and fidelity of various proteins. Around one-third of AML patients show frameshift mutations in nucleophosmin 1 (NPM1), affecting its role in mRNA processing, ribosome biogenesis, and chromatin remodeling [137]. These mutations alter NPM1, causing it to localize in the cytoplasm [138,139]. This altered localization may connect to decreased 2′-O-methylation guided by C/D box snoRNAs in AML cases with NPM1 mutations [140] (Figure 5). Npm1 inactivation in adult HSCs results in bone marrow failure. SnoRNAs like SNORD15, SNORD47, and SNORD104 have been shown to play roles in pathogenesis: their inactivation reduces colony formation, while the inactivation of SNORD15, SNORD47, SNORD52 and SNORD58 boosts erythroid cell differentiation. These data demonstrate that individual snoRNAs may have distinct roles in specific biological processes [140].

b. *snoRNAs in neurodegenerative disorders*

Box C/D snoRNAs are linked to neurodegenerative diseases, particularly Prader–Willi syndrome (PWS) [141,142,143], caused by a loss of paternal genes on chromosome 15. PWS features include mental retardation, short stature, obesity, and muscle weakness, and involves box C/D SNORD115 and SNORD116 [141,143]. In PWS mice lacking SNORD115, increased editing of serotonin 2C receptor (5HT_2c_R) pre-mRNA occurs without significant splicing changes [141]. This altered RNA editing, resulting from SNORD115 loss, may contribute to PWS symptoms. SNORD116 loss is also believed to play a major role in PWS development [143,144]. In mouse models of PWS, deleting the SNURF-SNRPN locus alters pre-mRNA splicing patterns. SNORD115 and SNORD116 can form shorter snoRNAs (psnoRNAs), influencing pre-mRNA splicing. SNORD115 generates several smaller RNAs, e.g., B-115, C-115, D-115 and E-115 forms referred as psnoRNAs, and these act to regulate the alternative splicing of several pre-mRNAs [113,145]. There is a debate about the prevalence of psnoRNAs in human and mouse brains; some view PWS snoRNAs as typical box C/D snoRNAs [146]. SNORD115 and SNORD116 dysregulation is noted in Alzheimer’s disease (AD) as well, affecting RNA processing and protein synthesis, leading to neuron dysfunction and cognitive decline [147,148,149,150]. The effect of SNORD115 on RNA processing possibly influences Aβ production and clearance, and tau protein regulation in AD [151]. Studies have linked SNORA74A, which regulates BACE1, to Aβ peptide production, a key factor in AD, and its dysregulation may lead to Aβ accumulation [152]. SNORD115 dysregulation is also linked to brain development issues and behavioral abnormalities (viz. poor social interaction, behavioral inflexibility, abnormal ultrasonic vocalizations or communication, and anxiety) in autism-related mouse models [153].

Furthermore, the dysregulation of snoRNAs like SNORD118 and SNORA7B is observed in Parkinson’s disease (PD) [154,155]. SNORD13, for instance, is specifically associated with Huntington’s disease (HD), involved in genomic activities related to HD pathophysiology [156], with increases plasma levels indicating disease progression. Therefore, snoRNAs can play a crucial role in the pathogenesis of neurodegenerative disorders, impacting RNA processing and protein synthesis in neurons, essential for neuronal health.

c. *snoRNAs in solid cancers*

Box C/D snoRNAs are linked to the development of various cancers. SNHG5, a snoRNA host gene, contains SNORD50, which is associated with beta-cell lymphoma and solid tumors [157,158]. Significantly, 10–40% of common cancers have somatic deletions in SNORD50A and SNORD50B genes, indicating their possible involvement in cancer formation [159]. SNORD50A and SNORD50B normally inhibit Ras oncoproteins, including KRAS [159]. Their loss results in higher active KRAS and Ras-ERK1/2 signaling, which encourages tumor growth in CHL-1 parental melanoma cells. Additionally, deleting these snoRNAs in cells with a KRAS mutation, often found in cancer, further promotes tumorigenesis [159].

Research using RNA-Seq and RT-qPCR in 106 prostate cancer cases showed increased snoRNA-derived RNAs levels in metastatic disease [78]. High levels of the box C/D snoRNA SNORD78, and its derived RNA, sd78-3′, are linked to severe prostate cancer metastasis [78]. Moreover, SNORA55 is crucial for cell proliferation and migration in prostate cancer, with its expression closely linked to prognosis and TNF-GHRH-dependent oncogenic pathways [160]. Other snoRNAs like SNORA73A, SNORA73B, and SNORA74A, bind to PARP-1, influencing ribosome biogenesis and breast cancer growth by activating RNA Helicase (DDX21) through ADP ribosylation [161] (Table 1). Their exact mechanisms need additional research. Also, SNORD33, SNORD66, and SNORD76 are highly expressed in non-small-cell lung cancer (NSCLC) patients’ plasma, serving as sensitive and specific markers for NSCLC [162], which causes about 25% of cancer-related deaths. GeneChipR Oligo arrays studies have linked SNORA42 and SNORD78 with NSCLC [163,164].

Small derived RNAs (sdRNAs) from snoRNA loci may significantly impact cancer. A high expression of SNORD6 is implicated in cervical cancer, one of the most common cancers in the female reproductive system [165], and is often associated with human papillomavirus (HPV) infection. SNORD6 knockdown leads to reduced cell cycle arrest, decreased cell migration and invasion, and increased apoptosis [165]. It is involved in tumor-suppressor protein p53 degradation by forming the E6-E6AP-p53 complex, aiding in cell cycle progression and proliferation [165]. Further, SNORA21 and SNORA42 are identified as major risk factors for colon or gastric cancer [166]. These findings enhance our understanding of basic research on malignant tumors and could provide new perspectives on diagnosing and treating these cancers.

**Table 1:** Some therapeutically potent snoRNAs involved in different cancers/disease and their mechanism.

Disease	SnoRNA/Target	Signaling	Mechanism
Breast cancer and NSCLC [167,168]	U3 or U8/ Ribosomal proteins uL5 (RPL11) and uL18 (RPL5)	Antitumor protein p53 is upheld at a low level by constitutive polyubiquitination by the E3 ubiquitin ligase Hdm2, followed by proteasomal degradation	Inhibits p53-dependent anti-tumor stress response involving the ribosomal proteins, and involved in ribosome biogenesis, nucleolar structure, and tumorigenesis.
Breast cancer, and microcephalic osteodysplastic primordial Dwarfism type I (MOPD1) [169]	SNORD28/ TAF9B (p53 coactivator)	p53-related pathway	SNORD28 undergoes additional processing to produce the miRNA sno-miR-28. TAF9B, a p53 coactivator, was suppressed by this miRNA.
Breast and ovarian cancers, and other cancer types [161]	SNORA73A, SNORA73B, and SNORA74A/ PARP-1	PARP-1-related DNA repair and gene regulation	These snoRNAs bind to PARP-1 and stimulate PARP-1, activated PARP-1 PARylates the DDX21 to promote nucleolar localization and they involved in ribosome biogenesis
Melanomas, ovarian, liver, lung, and breast cancers [159]	SNORD50A and SNORD50B/K-Ras	Ras-ERK1/ERK2 signaling	Absence of SNORD50A and SNORD50B increases the amount of GTP-bound, actives K-Ras and hyperactivated Ras-ERK1/ERK2 signaling and increases binding by farnesyltransferase to K-Ras and K-Ras prenylation.
Ovarian cancer [170]	SNORA72/Notch1-c-Myc; Nanog, Oct4	Notch1/c-Myc pathway	SNORA72 elevates the expression of Notch1 and c-Myc proteins along with CD133, Nanog, and Oct4 proteins.
Leukemia [136]	SNORD34, SNORD35A, SNORD43, and SNORD104/Associated rRNA	rRNA methylation	These snoRNAs’ deletion disrupts the methylation of rRNA, reduces cell volume, and slows the rate at which damaged proteins are repaired.
Acute lymphoblastic leukemias [171]	SNORD112–114/Rb	Rb/p16 pathways	Expression of these snoRNAs affects Rb/p16 cell cycle regulation.
HBV-related hepatocellular carcinoma [172]	SNORA18L5/p53	MDM2-mediated ubiquitination and degradation of p53	Germline duplication of SNORA18L5 causes alterations in the localization of Ribosomal proteins (RPL5 and RPL11) and decreases the P53 level.
Hepatocellular carcinoma [173]	SNOU2_19/β-catenin	Wnt/β-catenin signaling pathway	Promotes the translocation of β-catenin in cytoplasm inhibiting apoptosis and inducing cell cycle progression.
Hepatocellular carcinoma (HCC) [174]	SNORD52/CDK1	Upf1-SNORD52-CDK1 pathway	SNORD52 combines with CDK1 and increases its protein level by improving its stability.
Glioblastoma [175]	SNORD76/Rb	Rb-associated cell cycle	Through altering the regulation of the Rb-associated cell cycle, snoRNA76 prevents glioma cells from being tumorigenic.
Human pancreatic ductal adenocarcinoma [176]	SNORA23/SYNE2	Ribosome biogenesis	It enhances mRNA and protein expression of spectrin repeat-containing nuclear envelope 2 (SYNE2).

d. *snoRNAs in stress response and metabolic homeostasis*

Metabolic stress activates adaptive responses in cells to survive under harsh conditions. This includes mechanisms that preserve cellular functions during critical illnesses or tough environments. A key trigger is lipotoxicity, caused by excess lipid uptake, especially saturated fatty acids. This affects the endoplasmic reticulum (ER) membrane, leading to organelle damage and an ER stress response [177,178]. Similar changes in mitochondria cause dysfunction, disrupting energy production and inducing programmed cell death [179,180]. These processes result in reactive oxygen species (ROS) production, which activates pro-inflammatory cytokines transcription through NF-κB, triggers NADPH oxidase, and stimulates death receptor signaling [181,182,183].

Various research groups have identified key regulators of metabolic stress responses, primarily enzymes. Additionally, non-coding RNAs like lncRNA, Gadd7, and ribosomal protein L13a (Rpl13a) locus-encoded box C/D snoRNAs are involved in managing the response to lipotoxicity [184,185,186]. Studies indicate that losing Rpl13a function enhances resistance to lipotoxicity and metabolic stress [184]. The deletion of Rpl13a snoRNAs changes mitochondrial metabolism, lowers ROS levels, and increases glucose-stimulated insulin secretion from pancreatic islets, thus improving systemic metabolic homeostasis related to glucose regulation [187].

SNORA73A and SNORA73B also play key roles in metabolic stress responses by regulating cell metabolism through the mTOR pathway. Deleting SNORA73 restructures cellular metabolism and reduces steatohepatitis [188]. In lipotoxic conditions, superoxide induction is followed by increased cytosolic snoRNA, with superoxide levels directly affecting cytosolic snoRNA expression. Changes in superoxide dismutase and other superoxide inducers raise ROS levels, causing Rpl13a snoRNAs to translocate to the cytosol. Apart from their main function in ribosomal RNA modification within the nucleolus, Rpl13a snoRNAs are also crucial in cell death due to lipid overload [184].

Moreover, protein kinase RNA-activated (PKR), a stress response kinase, phosphorylates eukaryotic initiation factor-2 (eIF2α), inducing cell death in response to cell damage [189,190]. PKR activates not only eIF2α but also other proteins involved in inflammatory signaling, like Jun N-terminal protein kinase and IκB kinase [191]. In obese mice, PKR deletion or chemical inhibition improves glucose metabolism. Under metabolic stress, wildtype PKR interacts with snoRNAs, especially SNORA3, SNORA71, and SNORD113, which are crucial for PKR activation [192]. Further, eIF2α phosphorylation results in forming non-membranous stress granules, containing non-translating mRNAs and various proteins, aiding cell survival in stressful conditions [193]. Conversely, osteoarthritis can result from a disruption in metabolic homeostasis, causing changes in chondrocyte phenotype. SNORD116 knockout mice show reduced bone formation, linking the Prader–Willi critical region to osteoporosis [194]. Changes in the expression of SNORD116, SNORD26, and SNORD96A affect the expression of chondrogenic and hypertrophic ribosomal RNA, as well as genes associated with osteoarthritis [195].

e. *snoRNAs in infectious diseases*

We further discuss how viral infections activate the transcription of immune-related genes that inhibit virus replication and transcription. While the exact functions of many snoRNAs in viral contexts are not fully understood, their potential involvement and impact on viral infections are discussed.

Research shows that snoRNAs can be exploited by viruses to regulate their life cycle and suppress the host’s antiviral response. Most viruses do not produce their own snoRNAs, except for the Epstein–Barr herpesvirus (EBV), which causes mononucleosis and encodes a virus-specific v-snoRNA1. This v-snoRNA1, processed into smaller RNAs like miRNAs, is highly upregulated during infection, but its deletion showed no clear phenotype [196]. Gene-trap insertional mutagenesis experiments have provided evidence of SNORDs (C/D box snoRNAs) and SNORAs (H/ACA box snoRNAs) in viral infections. In studies on 12 different viruses using cell lines with gene trap libraries, 83 SNORDs and SNORAs were detected—4 as independent transcription units and 79 within the introns of 29 genes [197]. The non-protein coding SNHG1 transcript hosts eight SNORDs significantly impacting all viruses studied. Among these, three SNORDs have their own promoters, suggesting their essential role, but not as hosting genes, in replicating various DNA (CPV, HSV2) and RNA (DFV, FLU, HRV16, and RSV) viruses [197]. This relationship between viruses and SNORDs is noted in multiple individually studied viruses.

Researchers used microarray and qPCR to analyze the snoRNA signature in HEK293T cells infected with Chikungunya virus (CHIKV) [198]. They observed increased levels of C/D cluster snoRNAs, especially U3, U44, U76, and U78. CHIKV infection also strongly induced TGF-β (SMAD6, JUN, SKIL) and endocytosis pathway (CXCR4, HSPA8, ADRB1) genes but reduced cell cycle genes (CDC27, CDC23). This suggests potential biomarkers and therapeutic targets for CHIKV infection [198]. Studies profiling snRNA types and quantities in blood have distinguished between healthy pigs and those infected with porcine reproductive and respiratory syndrome virus (PRRSV) [199]. Using transcriptomic read counts, researchers identified differences in microRNAs, small nucleolar RNAs, and tRNAs. These three RNA classes showed significant variations and may influence host dysregulation during PRRSV infections [199], suggesting potential areas for further investigation. Sequencing small RNA libraries from bovine kidney cell lines, both uninfected and infected with Bovine herpesvirus 1, suggests that some snoRNA loci may function similarly to miRNAs [200]. Additionally, a dual analysis of Murine Cytomegalovirus (MCMV) and host cell transcriptomes during lytic infection revealed many new spliced and unspliced MCMV transcripts. The top downregulated and repressed genes, including long intergenic ncRNAs, antisense RNAs, and snoRNAs, were linked to obscure infection-related functions [201].

RNA viruses, especially (+)ssRNA viruses like *Nidovirales,* which include coronaviruses, encode RNA processing machinery like the NendoU endonuclease. NendoU is like XendoU, a cellular endoribonuclease A from *Xenopus laevis* involved in snoRNA U16 and U86 maturation and other snoRNA processing activities [202]. Retroviruses often incorporate non-coding RNAs into their virions, like murine leukemia virus incorporating SNORD104 [201], although the mechanisms remain unclear. The functions of overexpressed SNORDs and other ncRNAs in HIV-1 and Maloney leukemia virus are also not fully understood [203,204]. An RNA sequencing analysis of A549 cells infected with H1N1 influenza virus identified 121 snoRNAs, but their specific roles in influenza virus infection are yet to be determined [205].

f. *snoRNAs in aging*

Cell failure occurs when cells reach a certain number of divisions, leading to an “irreversible” stagnation in their cycle—a concept known as cellular senescence, first proposed by Leonard Hayflick in 1961. Research shows that snoRNAs, specifically SNORD96A and SNORD44, play a critical role in cartilage aging [195]. Studies on senile osteoporosis, which is linked to the aging of bone marrow mesenchymal stem cells (BMSCs), indicate significant changes in 63 snoRNAs during BMSC senescence [206]. In Drosophila, mutations in a new snoRNA, jouvence, affect lifespan, with reductions shortening it and overexpression in enterocytes prolonging it [207]. In the human immune system, aging affects B cells’ ability to produce antibodies, impacting our response to viral infections and vaccinations. A study found increased expression of SNORD123 and Cdkn2a in elderly mice’s B cells compared to younger mice, suggesting B-cell aging as a contributing factor to diminished immune response in aging [208].

## 9. snoRNAs as Therapeutic Targets

It has become increasingly evident that snoRNAs have regulatory roles beyond housekeeping genes, impacting signaling pathways, tumorigenesis, apoptosis, metabolic stress, genetic disorders, and other physiological functions, making them potential therapeutic targets and/or diagnostic tools. SNORA71A is elevated in various lung cancers [209]; its downregulation in NSCLC cells causes cell cycle arrest and reduces migration and invasion [209]. SNORA72, when overexpressed in ovarian cancer cells, increases Notch1 and c-Myc proteins, affecting cell growth and migration [170]. Reducing SNORA72 decreases cell self-renewal and migration [170]. The expression of SNORA3 and SNORA42 was inversely associated with the survival of NSCLC patients; silencing SNORA42 in lung cancer inhibits tumor-initiating cell growth [210]. In hepatocellular carcinoma, SNORD52, regulated by Upf1, promotes tumorigenesis [174]. Targeting the Upf1-SNORD52-CDK1 pathway could be a potential treatment. In pancreatic cancer, high SNORA23 levels are linked to increased SYNE2 expression, and using its antisense oligonucleotides controls tumor growth [176]. In contrast, in glioblastoma, snoRNAs like SNORD76 suppress tumors, with overexpression reducing glioma growth by altering Rb, cyclin A1, and B1 expression [175], offering a potential therapeutic approach.

Moreover, SNORA73 downregulates the hypoxia-upregulated mitochondrial movement regulator (HUMMR), affecting cholesterol homeostasis and gonadal tissue maturation [211], and alongside SONRD60, it influences lipid metabolism, with its deletion protecting against steatohepatitis [188,212]. Recent studies reveal varied snoRNA expression in cardiovascular disease patients [213,214,215]. For instance, the DLK1-DIO3 locus shows high snoRNA levels in end-stage heart failure, compared to other tissues [216]. SnoRNAs can influence tRNA-derived fragment formation by preventing tRNA cleavage. Overexpressing angiogenin produces stress-like tRNA halves [217,218]. SNORD113-6, by methylating tRNA, protects against fragmentation in vascular remodeling, crucial in cardiovascular disease [219]; targeting it with antisense oligonucleotides or siRNAs may help in improving cardiovascular disease. Therefore, given their critical involvement in cardiac development, pathophysiology, and lipid metabolism, snoRNAs emerge as promising new candidates for therapeutic targets and biomarkers for disease [184,220].

## 10. Prospects; snoRNAs as Molecular Machinery

Originally identified as a defense mechanism in bacteria against certain bacteriophages, CRISPR-Cas9 has become a key tool in genetic and infectious disease research, including COVID-19 treatment. There is also a growing interest in using snoRNAs for the targeted biochemical modifications of molecules like mRNA, tRNA, or rRNA. Regulating snoRNAs is seen as particularly promising in molecular biology.

Many snoRNAs are highly expressed in diseases. These snoRNAs normally regulate cell growth. For example, SNORD50A, high in U/A content, controls gene expression by blocking mRNA 3′ processing, interfering with the Fip1-poly(A) site interaction [221]. Additionally, SNORA14A’s overexpression causes G2/M phase arrest, cell death, and reduced hepatoblastoma cell growth. This overexpression aids in processing 18S rRNA, increasing succinate dehydrogenase subunit B (SDHB) protein levels [222]. Notably, overexpressing SDHB leads to G2/M phase arrest and anti-growth, pro-death effects in cells. However, succinate stimulates hepatoblastoma cell growth in varying doses [222,223,224].

**Figure 6:**
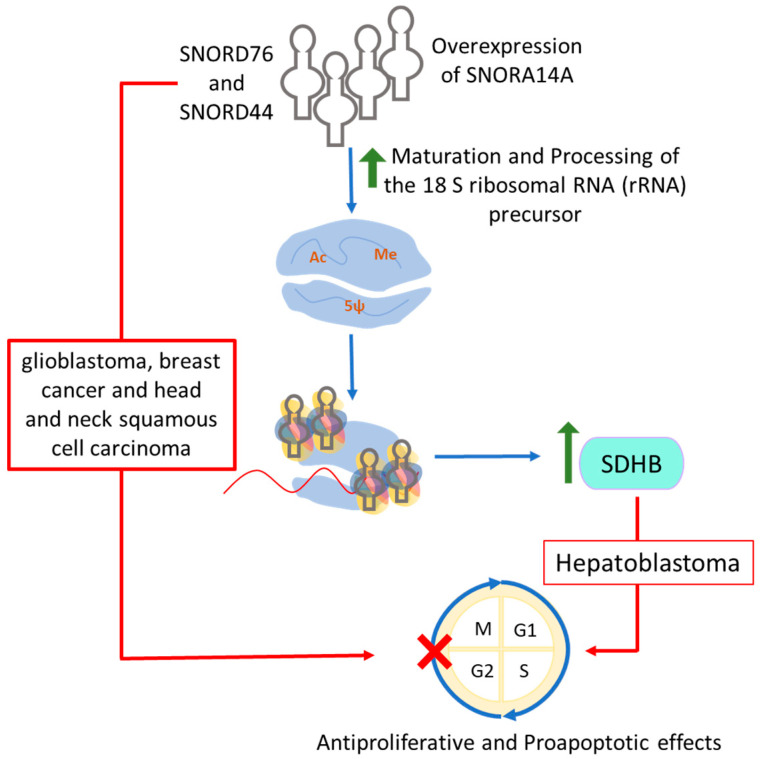
**Figure representing the anti-cancerous nature of some snoRNAs.** In hepatoblastoma, SNORA14A (H/ACA box snoRNA) is overexpressed and enhanced the ribosomal biogenesis required for the expression of succinate dehydrogenase subunit B (SDSB) protein. SDSB can reduce the cellular proliferation and exerts apoptotic effects in hepatoblastoma cells. Similarly, the C/D box snoRNAs, SNORD76 and SNORD44, also exhibit antiproliferative effect in multiple cancers.

Alternatively, one group shows that snoRNAs can have tumor-suppressing properties due to lower expression in diseases or cancer. SnoRNAs from GAS5, like SNORD76 and SNORD44, are less expressed in glioblastoma, breast cancer, and head and neck squamous cell carcinoma, indicating their role as tumor suppressors. Normally, these snoRNAs help inhibit cell proliferation (Figure 6) [175,225,226]. Given the current knowledge, it is reasonable to suggest that tumor-suppressive snoRNAs might soon be used as molecular tools to manage diseases or cancers, like how oligonucleotides with similar sequences to those snoRNAs can be used to increase the levels of these snoRNAs.

## 11. Conclusions

SnoRNAs play a vital role in RNA processing and impact different molecular pathways in a range of pathologies, but many of their specific functions are still unknown. In chronic metabolic diseases, these ncRNAs affect tumorigenesis, cancer progression, cell death, oxidative stress, inflammation, reactive oxygen species production, cholesterol metabolism, and diabetes. These snoRNAs exhibit dual roles as both oncogenic and tumor-suppressive agents, offering therapeutic targeting opportunities and the potential regulation of oncogenic gene expression. Although research is early, snoRNAs’ diverse roles are gaining scientific interest.
